# A Comparative Study on the Performance Profile of Under-17 and Under-19 Handball Players Trained in the Sports School System

**DOI:** 10.3390/ijerph17217979

**Published:** 2020-10-30

**Authors:** Tomasz Gabrys, Arkadiusz Stanula, Subir Gupta, Urszula Szmatlan-Gabrys, Daniela Benešová, Łukasz Wicha, Jakub Baron

**Affiliations:** 1 Department of Physical Education and Sport Science, Faculty of Pedagogy, University of West Bohemia, 301 00 Pilsen, Czech Republic; tomaszek1960@o2.pl (T.G.); dbenesov@ktv.zcu.cz (D.B.); 2 Institute of Sport Science, The Jerzy Kukuczka Academy of Physical Education, Mikołowska 72A, 40-065 Katowice, Poland; j.baron@awf.katowice.pl; 3 Faculty of Medical Sciences, University of West Indies, 11000 Cave Hill, Barbados; subir.gupta@cavehill.uwi.edu; 4 Faculty of Rehabilitation, Department of Anatomy, University of Physical Education, 31-571 Krakow, Poland; urszula.szmatlan@awf.krakow.pl; 5 Polish Handball Federation, Puławska 300 A, 02-819 Warszawa, Poland; lukaszwicha85@gmail.com

**Keywords:** power output, aerobic threshold, anaerobic threshold, peak blood lactate, jump tests

## Abstract

This study evaluates the anatomical profiles, jump, sprint, power outputs, endurance, and peak blood lactate levels ([LA]_peak_) of handball players of two age groups—U17 (*n* = 77) and U19 (*n* = 46)—and analyses the role of training in their physical abilities. Vertical jump performance was determined by counter movement jump (CMJ) and counter movement jump with free arms (CMJFA) tests. A running-based anaerobic sprint test (RAST) determined the relative power output (watts/kg body weight) and absolute power output (watts) of the players. Sprint performance over 5 m, 10 m, and 30 m distances was evaluated. An incremental shuttle run test (40 m) was designed to determine aerobic threshold (AeT), anaerobic threshold (AnT), and [LA]_peak_. All parameters were measured for pivots, wingers, backs, and goalkeepers of each group. The U19 players were significantly heavier than the U17 group, but both the groups were nearly equal in height. The U19 group jumped higher than the U17 members, although the only significant difference (*p* = 0.032) was observed between the wingers of the groups in CMJ. Sprint performance varied marginally between the groups and only U19 pivots were found to be significantly (for distances of 5, 10, and 30 m: *p* = 0.047, *p* = 0.018, and *p* = 0.021, respectively) faster than U17 pivots. No difference in relative power output between the groups was noted, although the U19 players recorded higher absolute power outputs. Maximal velocity and velocities at the AeT and AnT were almost similar in the groups. Distance covered by the groups at the intensities of AeT and AnT varied only little. Higher [LA]_peak_ was observed in the U19 players. U19 players failed to convert their superior power into speed and jump. The training pattern of the handball players needs to be revised so that U19 players may develop faster and be more enduring than the U17 group.

## 1. Introduction

Selection and training systems of sports schools in Poland, coordinated by various sports bodies, including the Polish Handball Federation, have merits and demerits. Besides providing sports training to their athletes, sports schools in Poland focus on other social matters as well. The assessment of a player’s talent in team sports is based on three areas: motor skills and physique, mental health, and social features [1,2,3]. A common training program in the sports schools has its pros and cons. A common physical training program in sports schools across the country is likely to cause similar physiological adaptation in the adolescents and young players, although individual development factor plays an important steering force in their overall development. However, a similar training pattern does not create opportunities to implement sports-specific training programs [4,5,6].

Handball players require specific training that allows them to perform cyclic and acyclic activities efficiently during 60 min of match play. Like many other team sports, the movement pattern of handball players during match play is intermittent, intense, and varies widely in phases of defense and attack [7,8]. Apart from physical training, the performance of a handball player is influenced by anthropometric, physiological, and kinematic factors, like many other team sports [9,10,11]. Match-specific fitness of a handball player can be evaluated by a number of well-designed field tests [12]. The physical and physiological characteristics of handball players of various levels have been studied extensively by researchers [13,14,15]. These are concerned with aerobic capacity, anaerobic endurance, and anaerobic power determined by sprint run, jump, and throw [13,14,15].

The differences between youth (16–19 year) handball players of various levels have been documented by researchers [16,17]. Handball matches are played at the intensity range of 65 to 85% VO_2max_ and at a blood lactate concentration of 3 to 11 mmol/L. The VO_2max_ of the youth (16–21 year) handball players varies from 50 to 65 mL/kg/min, which partly depends on their position of play. The blood lactate concentration in same group of handball players after a run ramp test for the exhaustion is 10–12 mmol/L [14,18,19,20,21,22]. The endurance capability of athletes is commonly assessed by measuring their performance or running speed at the level of anaerobic threshold (AnT), which reflects the running economy and efficiency of the player. The VO_2max_ reflects the endurance potential of a player and is not as important a marker of economy of run as the AnT [23,24]. In spite of the dominance of aerobic metabolism, the sport of handball is interspersed by high intensity activities like jumps, throws, changes of direction, and stops that greatly tax anaerobic metabolism [18,23]. The load imposed on the players is determined mostly by the demand of the game. Much of the movement during a handball game takes place in quick succession, with brief rest or slow movement in between. Although these activities are anaerobic in nature, the need to repeat them frequently demands a high level of aerobic capacity. Endurance training is designed to delay the appearance of fatigue of the players, during both training and match-play [19,25].

Handball is a game with a large number of explosive movements such as accelerations, turns, jumps, and throws [4]. Therefore, in assessing a player’s progress, it is very important to measure the anaerobic capacity of the player. Sprint, jump, and throw are commonly measured to assess the anaerobic power of a handball player [26,27,28]. Studies [29,30] show that CMJ values in the groups of handball players aged 16–19 do not show significant progression similar to the mean power in the RAST test [29,31]. However, to the best of our knowledge, no studies have been conducted so far that evaluated the physical and physiological profiles of age-group handball players in the sports schools of Poland. The age of 17–19 years in the development of handball players is a period of transition from junior handball to the requirements for handball players in senior teams, as indicated by the values of motor preparation indicators recorded in other studies [14,15,29,32]. It should be expected that, during this period, there will be a significant development of motor skills such as endurance, relative power, and running speed. The sports schools of Poland still follow the guidelines for the selection of players and their training set by the concerned authority decades before. A review of the selection criteria and training programs is absolutely essential, especially in the light of recent advancement of sports science and training. The aim of the present study is to revise the selection criteria and efficacy of training of the U17 and U19 handball players of the Polish Handball Federation Sports Schools, by comparing anthropometric profiles and explosive power, sprinting ability, and selected physiological characteristics.

## 2. Materials and Methods

### 2.1. Participants

A total of 133 male handball players, all of whom were students of the Polish Handball Federation Sports Schools at Gdańsk, Kielce, Kwidzyn, and Płock, participated in this study. Players were divided into two categories: U17 (age: 15 to <17 years) and U19 (age: 17 to <19 years). In each age group, the players were divided according to the position: pivots (U17: *n* = 7, U19: *n* = 10), wingers U17: *n* = 8, U19: *n* = 22, backs (U17: *n* = 26, U19: *n* = 42), and goalkeepers (U17: *n* = 5, U19: *n* = 13). All of the U19 and some of the U17 players were selected for the Junior Polish National Team. Body weight and height of the participants and their playing positions are presented in Table 1. All subjects (in the case of 18+ years) or their parents or legal guardians (in the case of <18 years) provided their written consent to participate in this study after being informed of all procedures and risks involved in this study. They were in good health and reported no injuries and infections at the time of the study. The study was conducted during a scheduled training week, before competitive session.

The whole experiment was divided in two sessions and conducted over two days. The two experimental sessions were separated by approximately 24 h. Subjects were instructed to refrain from all sorts of caffeine ingestion 48 h before tests. In the first session, all subjects performed a countermovement jump (CMJ) test and countermovement jump with free arms (CMJFA) test, sprint run over a distance of 30 m, and running-based anaerobic sprint test (RAST), after 20 min of break. Both the jump and the sprint tests were performed twice and only the better result was analyzed for this study. In the second session, the endurance capability of the subjects was evaluated. The endurance test and the RAST, however, were performed once only.

#### 2.1.1. Jump Tests

All the subjects performed two jump tests—(1) counter movement vertical jump without arm swing (CMJ): participants were instructed to stand upright comfortably with hands on hips. They remained in this position for 3 s before the jump was performed. Following a verbal command, the players initiated a countermovement followed by a maximal vertical jump in one continuous motion. Participants were instructed to keep their hands on their hips throughout the jump, and their legs straight while in the air [33,34,35]. (2) Counter movement vertical jump with arm swing (CMJFA): this differs from the CMJ in that both the arms were allowed to move freely during the vertical jump [36]. Each of the jump tests was repeated twice, with a passive recovery of 1 min between the two, and the best result was recorded and analyzed. Optojump (Microgate, Srl., Bolzano, Italy) was used for the measurement of the jump height.

#### 2.1.2. Sprint Test

This test was preceded by a non-standardized 20 min warm-up. Subjects performed two 30 m sprint runs starting from a standing position. A 4 min interval with light active recovery separated two trials. Time was recorded using SMARTSPEED PRO time gate system (Fusion Sport, Brisbane, Australia). Time was measured at the 5 m, 10 m, and 30 m marks. Time measured for the 5 m and 10 m distances indicated the ability to quick start, whereas the speed achieved for the 30 m distance reflected the speed usually obtained during the transition from defense to attack phase in typical handball match play.

#### 2.1.3. Running-Based Anaerobic Sprint Test (RAST)

Anaerobic capacity was measured by a running-based anaerobic sprint test (RAST). It has been shown that this test can replace the Wingate test to estimate anaerobic power and capacity [37]. Each subject completed six 35 m sprint runs, with 10 s passive rest between two repetitions. Time was recorded using SMARTSPEED PRO time gate system (Fusion Sport, Brisbane, Australia). The power output (in watts) for each sprint was calculated according to the following equation:

Anaerobic capacity (Watt) = Weight (kg) × Distance (m) ^2^ ÷ Time (s) ^3^ (1)

Fatigue Index = [Maximum power (watts) − Minimum power (watts)] ÷ Total time (s) for the 6 sprints(2)

The RAST gives an estimate of the neuromuscular and energy determinants of maximal anaerobic performance and is a simple, but very useful test used in team sports like handball, where running is an important component of activities [38].

#### 2.1.4. Endurance Test

This is a multistage fitness test that determines endurance by an incremental shuttle run. This test was conducted on a synthetic surface in an indoor hall. The test involved continuous running between two lines 40 m apart [39]. Subjects started running at the speed of 8 km/h, which is increased by 1.5 km/h after every 3 min thereafter, until exhaustion. To assure the constant running speed, subjects were instructed to adjust their pace using audio signals. The audio signal was given at the end and at the middle of 40 m distance. The end of the test was considered when the participant twice failed to reach the front line in time (objective evaluation) or he felt not able to cover another shuttle at the dictated speed (subjective evaluation) [40].

During the test, heart rate was continuously recorded at an interval of 5 s using Polar Team2 Pro chest-worn heart rate monitors (Polar Electro, Kempele, Finland). Maximal heart rate (HR_max_): the highest heart rate (HR) recorded at the exhaustive stage of the endurance test was considered as the HR_max_.

Collection of blood samples for the measurement of lactate concentration: after the end of each stage of run, the subjects were stopped for 30 s, during which 20 mL of capillary blood was collected from earlobe by pin-prick under aseptic conditions. Blood samples were also taken at the end of the test and at the 4th and 8th minutes of the recovery period. Blood lactate concentration ([LA]) was measured using a lactate analyzer (Biosen C-Line EKF Diagnostic GmbH, Magdeburg, Germany).

Peak blood lactate concentration ([LA]_peak_): the highest [LA] recorded following the end of the test was recorded as the [LA]_peak_.

Determination of aerobic threshold (AeT): the running intensity at which the [LA] increased by 0.5 mmol/L was marked as the AeT [41].

Determination of anaerobic threshold (AnT): the running speed at the [LA] of 4 mmol/L was considered as the AnT of the player [42]. 

The total distance of run, completed by the subject in the endurance test, was divided into three intensity zones—(a) zone 1: up to AeT, (b) zone 2: above AeT to AnT, and (c) zone 3: above the AnT level.

### 2.2. Statistical Analyses

Mean and standard deviation were used to represent the average and the typical spread of values of all the measured variables. The normal Gaussian distribution of the data was verified by the Shapiro–Wilk’s test. If the data were normally distributed within groups, an independent samples t-test was used to test the differences between U17 and U19. If the data were not normally distributed, a Mann–Whitney U-test was used. Two separate one-way analyses of variance with a Tukey post-hoc test were used to determine whether and where differences existed in the all measured variables between the playing positions in each age group. The effect size (ES) of the intervention was calculated using Cohen’s guidelines. Threshold values for ES were >0.2 (small), >0.6 (moderate), >1.2 (large), and >2.0 (very large) [43]. Statistical significance was set at *p* ≤ 0.05. All calculations were performed with STATISTICA ver. 13.3 (TIBCO Software Inc., Palo Alto, CA, USA).

## 3. Results

### 3.1. Body Weight and Height

Body weight and height of the U17 and U19 players are presented in Table 1. Weight and height between the U17 and U19 players for all position of play are compared. No statistically significant difference in body height between U17 and U19 players was noted. Under 19 players, however, were heavier than their U17 counterparts by 9.2 to 13.8% and the difference was significant in all cases, except in pivots.

### 3.2. Vertical Jump Performance 

**Figure 1:**
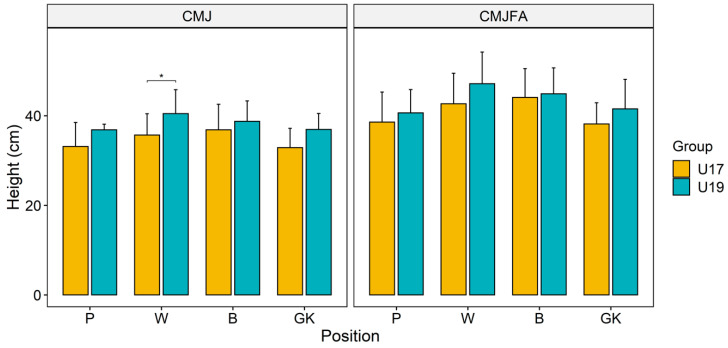
Test results of counter movement jump (CMJ) and counter movement jump free arms (CMJFA) of the participants. (P—pivots; W—wingers; B—backs; GK—goalkeepers; * *p* < 0.05).

Figure 1 presents the jump performance of the players. It compares the performance of the U19 and U17 players of each playing position. Superior jumping skill was demonstrated by the U19 players compared with the U17 players, for any given position of play, although the difference was non-significant in most of the cases. Wingers of the U19 group demonstrated significantly higher CMJ ability than the U17 players. As expected, CMJFA score was higher than all the respective cases of CMJ.

### 3.3. Sprint Performance

Sprint performance (time) of the participant handball players, for various distance marks, as well as differences in performance between U17 and U19 groups, and the *p*-values and the ES of the difference, are presented in Table 2. The players of all positions of the U19 group outperformed the respective U17 players in both the 10 m and 30 m sprint runs, although the difference was significant only in the case of pivots. On the other hand, except in the case of pivots, the U17 group members in the 5 m sprint demonstrated better performance than their U19 counterparts.

### 3.4. Running-Based Anaerobic Sprint Test (RAST)

Maximum power (P_max_), minimum power (P_min_), average power (P_av_), and fatigue index (FI), recorded in RAST, are presented in Table 3. All the power outputs are expressed in watts/kg body weight in this table. All the power outputs—P_max_, P_min_, and P_av_—of U19 outfield players exceeded the power outputs of U17 players of similar positions, although the difference in none of the cases was found to be significant. Only goalkeepers of the U17 group demonstrated higher P_min_ and P_av_ than the U19 goalkeepers, although the ES was only trivial. Wingers of both the U19 and U17 groups showed higher P_max_ and P_av_ than other players of their own group. No significant difference of FI between the groups was found and the ES varied from trivial to moderate.

Table 4 shows the power output of the players determined by RAST, but the power output in this table is expressed in watts. All the power outputs (P_max_, P_min_, and P_av_) of the older age group (U19) were recorded higher than those of the younger group (U17) of handball players and the difference was significant in all the cases, except P_min_ between the goalkeepers. The ES varied from moderate to large.

### 3.5. Endurance Performance

**Figure 2:**
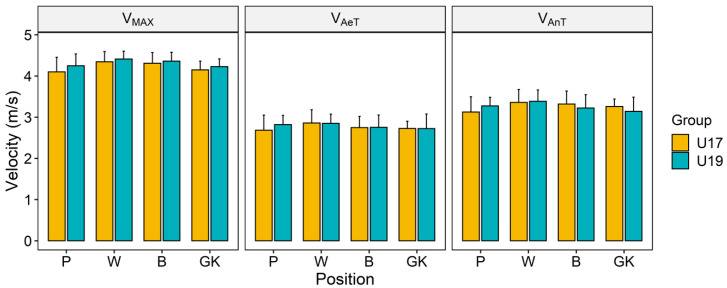
Velocity of the players at three intensity zones.

Maximum velocity (V_max_), velocity at the aerobic threshold (V_AeT_), and the velocity of the players at the intensity of anaerobic threshold (V_AnT_), determined in endurance test, are presented in Figure 2. The U19 players of most of the playing positions showed higher V_max_, V_AeT_, and V_AnT._ When compared between the age groups (U19 and U17), the difference of the velocity for any given intensity was only marginal, no significant difference was observed in any of the cases, and the ES varied only from trivial to small.

**Figure 3:**
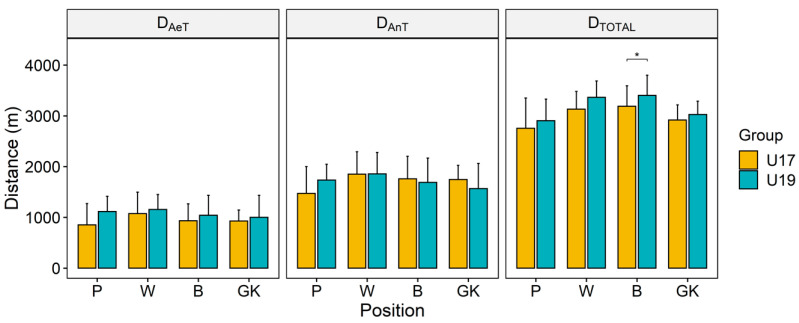
Distance covered by the players at different intensity zones in the endurance test.

The distance covered by the players at three different intensity zones is shown in Figure 3. The total distance covered (D_TOTAL_) is the arithmetic sum of the distance covered up to the aerobic threshold (D_AeT_) and the distance covered up to the intensity of anaerobic threshold (D_AnT_) (but higher than the AeT level). Like velocity, the difference of distance covered for any of the three given intensities between the groups (U19 and U17) was marginal again. Only D_TOTAL_ of the U19 backs was found to be significantly higher than that of the U17 backs.

Table 5 shows HR_max_% at the intensities of AeT (HR_AeT_) and AnT (HR_AnT_) of the participants. No significant difference in HR_AeT_ and HR_AnT_ between the age groups was found, except the HR_AeT_ of the backs.

**Figure 4:**
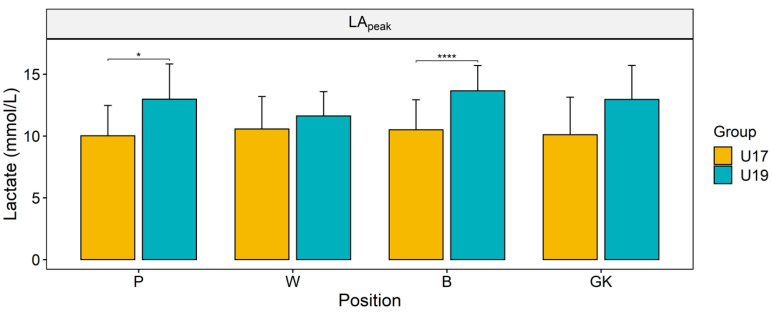
Peak blood lactate [LA]_peak_ concentrations in the participants determined in the endurance test (* *p* < 0.05, **** *p* < 0.001).

Figure 4 presents [LA]_peak_ of the subjects recorded after the end of the endurance test. The players of all the positions of U19 demonstrated higher [LA]_peak_ than their U17 counterparts, although the difference was statistically significant only between the backs (*p* < 0.001) and the pivots (*p* = 0.037) of the two groups.

## 4. Discussion

Handball players need specific training that allows them to do multiple and complex physical tasks successfully. Like many other team sports, age, training, skill, and playing position serve important roles in developing efficiency in handball players [8,44]. The requirements for playing in each position are determined by the appropriate physique, absolute power, and physiological profile of the player [45]. This, in turn, sets appropriate training demands for players of each playing position, and thus can differentiate a winger from a pivot or a back [15,25,46]. The key findings of this study are that the explosive power of the U19 players was superior to that of the U17 players, which resulted in better jump performance, but failed to improve speed in the U19 group. The training program in the sports schools of Poland was unsuccessful in the improvement of endurance in U19 players.

### 4.1. Pivots

Pivots experience more physical confrontations than players of any other positions, against the opponent team members, during a game. As a result, strength and explosive power are some of the primary requirements for pivots [6,29]. This is evident from the body weight and height of the U19 and U17 groups, where U19 pivots are nearly 10% heavier than pivots of the U17 group, but the difference in height between the two is almost absent (~1%). Another important requirement for pivots is the ability to run fast on a longer available space of the court (e.g., the 15–30 m segment of the sprint test in this study), which is especially very useful in counterattack. Fast attack and defense in quick succession, jumps, and powerful throws during game require appropriate anaerobic training of the players, especially for pivots [47]. A high training load elevates the serum growth hormone and testosterone in puberty and adolescence, which modulate muscle development and power.

Training of high intensity and long duration act as appropriate stimuli that favor to improve or maintain body stature and power [48]. Raspberry and Bouchard [49] have shown that resistance training can be carried out to a maximum load of 80% without appreciable risk of injury at this age. According to Gorostiaga et al. [50], a training program including high resistance exercises with slow movements that favor muscle hypertrophy hinders the development of explosive power, and thus favors sprinting ability. There was no difference in explosive power between the U17 and U19 groups as reflected by CMJ and CMJFA performance. Pivots of the U19 group showed significantly higher sprinting performance than the U17 pivots for all the distance marks (5 m, 10 m, and 30 m). This change of direction ability in pivot players was also reported in other studies [47,51]. The ability of change of direction with maximum power in pivots largely occurs between the ages of 16 and 19 years [52]. In all power ratings, U19s dominate over U16s. This is particularly evident when the power output was expressed in watts/kg body weight [31,38].

In case of the pivots, the anaerobic power generated was largely transferred to external loads, as noted by Krüger et al. [19]. This is partly supported by the fact that the [LA]_peak_ of the pivots of U19s was ~23% higher than that of the younger (U17) group of players. However, the V_max_, V_AeT_ and V_AnT_ of the U19 pivots were only marginally higher than those of the pivots of the U17 group.

### 4.2. Wingers

Jump, sprint; explosive strength; ability to accelerate, especially between 20 and 30 m distance; and reasonably high aerobic power are some of the key requirements for successful wingers [51,53]. There was no difference in body height between the age groups, although weight increased significantly (13.8%, *p* < 0.001; ES = very large) in the higher age group (U19). With a significant increase in body weight, a significant increase in anaerobic power and explosive strength is expected in U19 wingers compared with their U17 counterparts. However, an increase in muscle mass in U19, if any, and its role in the improvement of explosive strength requires further study. In the studied groups, no significant difference in speed over the distance of 5 to 10 m was found. The difference in explosive power is clearly visible in the CMJ results (*p* = 0.032) between the two age groups. The height of CMJ is higher by 11% and CMJFA by 9% in the U19 group. Thus, the increase in mass is accompanied by an increase in explosive power, but the increase in muscle mass did not support faster running in the U19 players. This observation is also supported by the fact that the absolute power (watts) output in the U19 players in RAST was significantly higher than that in the U17 group, but no such difference existed when the power was expressed in watts/kg body weight. While an increase in muscle mass is usually associated with an increase in power, there is no real difference in power with respect to the body mass in both age groups in this study. Improper training methods and loads probably failed to stimulate the relative increase in power in U19 players [54]. This was observed in other team sports as well [55,56]. The inclusion of agility in speed training brings much better results than repeated speed training or interval training in handball players [54,57,58]. Running economy in handball players, like many other team games, plays a crucial role in maintaining the higher intensity of work during match play without appreciable fatigue [59,60].

Running intensity above the AnT level resulted in more dependence on the anaerobic metabolism and decreased running economy of the players. The U19 players, except pivots, covered a longer distance above the AnT and produced higher [LA] than the participants of the U17 group. This suggests a decrease in the running economy in the U19 group compared with the U17 group [59]. The relationship between anthropometric parameters, including body weight and running speed, was pointed out by Kukoli et al. [61] and Young et al. [62]. The increase in absolute power generation without any change in relative power likely results from the increase in body weight of U19 players. The reason behind the lack of improvement of relative power in U19 players needs further study.

### 4.3. Backs

One of the major requirements of backs is a high level of strength combined with muscle mass. The U19 backs demonstrated higher glycolytic capacity than the backs of U17 group, as reflected by the 23% higher [LA]_peak_, in spite of lower HR_AeT_ in comparison with the U17 backs. The strength training is likely to improve the sprint, acceleration, and jumping and throwing abilities of the handball players. The longer distance covered by the backs during competitive match play needs reasonable aerobic training as well [19,53,63]. A significant difference in body weight (9%, *p* = 0.001; ES = moderate) of backs between the groups (U19 and U17) is noticeable, although no differences in explosive power and running speed were found between the groups in this study. No significant motor development beyond the age of 17 years is commonly found that would differentiate the U19 group from the U17 group, and this can explain why there is no significant difference in explosive power and running speed between the groups [47]. In backs, like pivots and wingers, power development in U19 players mainly results from an increase in muscle mass. A 10% increase in power is not enough to improve the speed significantly in U19 backs when compared with the backs of the U17 group. The total distance covered (D_Total_) by the backs of the U19 group exceeded the D_Total_ covered by the backs of the U17 group by 6%, mainly due to a much higher (>16%) D_AnT_, in spite of the lower D_AeT_. This again explains superiority of the anaerobic power in U19 backs in spite of compromised aerobic capacity.

### 4.4. Goalkeepers

Besides game-specific skill, handball goalkeepers are trained for developing explosive power, which is a prerequisite to efficient and effective jumping, throwing the ball, and quick acceleration of movement in all possible directions [19,27,32].

The height of the goalkeepers of both the age groups, like outfield players, does not vary significantly [64]. However, U19 goalkeepers were heavier than their U17 counterparts. Probably, stronger muscles of the U19 members were responsible for their superior performance in CMJ and CMJFA when compared with the U17 group. In spite of an increase in the explosive power of leg muscles, which improved their jumping ability, the U19 players were not faster than the U17 group. Higher FI in the U19 group suggests that they were unable to maintain the desired speed in repetitive sprints in comparison with the U17 goalkeepers. Longer D_Total_ and D_AeT_ by the U19 participants indicate higher aerobic capacity in U19 handball players than U17 players. More intense and frequent anaerobic training stimulated the anaerobic glycolytic system in the older group (U19) of handball players, and this was reflected by higher [LA]_peak_ in these players than the U17 participants. The reason for the limitations of the motor development of goalkeepers of U19 may be slowing down of biological maturity after 17 years of age [65].

## 5. Conclusions

The aim of this cross-sectional study was to compare physical fitness and some physiological characteristics of U17 and U19 Polish handball players with special reference to their position of play. Players of both the groups were equally taller, but the U19 members were significantly heavier than the U17 participants. Players of all the U19 positions dominate over the U17 players in terms of absolute power. However, a higher body weight has eliminated these differences in relative power values. Increases in body weight and total muscle mass in U19 players were responsible for superior explosive power, which caused better performance in the jump tests (CMJ and CMJFA) when compared with U17 players. The players of the U19 group showed lower running efficiency up to AeT level and longer distance covered above AeT than the U17 group. However, the U19 players possessed higher anaerobic capacity and an efficient glycolytic system compared with the group of U17 players. Players with higher body weight (U19) worked at a significantly higher energy cost level compared with the players of lower body weight (U17) of similar playing positions.

## 6. Study Limitations

The research has limitations on its wide use in men’s handball. The training system in a sports school has strict conditions that are different from training in sports clubs. The sports school players who participated in the research did not differ in the organization of the day, diet, and training loads. When relating the results of the presented studies to the values obtained in other training groups, the above limitations, which constitute an integral part of each sports training process, should be taken into account.

## 7. Practical Recommendations

Training of U19 groups should be targeted to transfer power into speed. There is a clear increase in maximum power with a lack of adequate development of running speed at distances important in handball (5 and 10 m). This is because of the fact that weight gain is not accompanied by an increase in relative power, so the observed power development is only compensated for by weight gain. It is also important because of the size of players, which limits their flexibility and ability to change directions rapidly. Development of endurance and running economy of the players should not be ignored, while focusing the training on the explosive power and sprinting ability of the players. It is recommended to increase the training impact at the age of 17–19 towards the development of running economy and speed increase on the thresholds (AeT and AnT).

## Figures and Tables

**Table 1:** Physical characteristics of handball players across their playing positions.

Indicator	Position	U19	U17	Mean Difference (%)	*p*-Value	Effect Size
Weight (kg)	P	101.9 ± 11.3	92.4 ± 14.93	9.52 (9.3%)	0.176	0.70/Moderate
	W	80.5 ± 3.10	69.4 ± 4.89	11.13 (13.8%)	<0.001	2.47/Very large
	B	86.5 ± 10.92	78.5 ± 8.71	7.99 (9.2%)	0.003 ^#^	0.83/Moderate
	GK	92.8 ± 11.69	81.6 ± 8.07	11.15 (12.0%)	0.033	1.22/Large
Height (cm)	P	190.3 ± 4.89	192.3 ± 6.06	−2.01 (−1.1%)	0.478	0.36/Small
	W	183.4 ± 4.07	180.5 ± 7.48	2.92 (1.6%)	0.307	0.43/Small
	B	188.7 ± 5.60	187.0 ± 6.68	1.71 (0.9%)	0.281	0.27/Small
	GK	189.6 ± 5.32	188.3 ± 4.64	1.29 (0.7%)	0.554 ^#^	0.27/Small

Note: P—pivots; W—wingers; B—backs; GK—goalkeepers; ^#^ a nonparametric test was used to compare the groups.

**Table 2:** Sprint performance of the U17 and U19 participant handball players.

Distance (m)	Position	Sprint Time (Seconds)		Mean Difference (%)	*p*-Value	Effect Size
		U19	U17			
5 m	P	1.06 ± 0.04	1.10 ± 0.03	−0.03 (−3.3%)	0.045 #	1.05/Moderate
	W	1.08 ± 0.05	1.05 ± 0.05	0.03 (2.7%)	0.151	0.61/Moderate
	B	1.05 ± 0.03	1.05 ± 0.04	0.00 (0.4%)	0.717	0.11/Trivial
	GK	1.10 ± 0.03	1.08 ± 0.06	0.02 (1.5%)	0.574	0.29/Small
10 m	P	1.80 ± 0.07	1.89 ± 0.06	−0.09 (−4.72%)	0.017 #	1.32/Large
	W	1.77 ± 0.07	1.78 ± 0.07	−0.01 (−0.6%)	0.721	0.15/Trivial
	B	1.76 ± 0.04	1.78 ± 0.07	−0.02 (−1.1%)	0.18	0.34/Small
	GK	1.83 ± 0.07	1.84 ± 0.08	−0.01 (−0.8%)	0.721	0.18/Trivial
30 m	P	4.38 ± 0.13	4.62 ± 0.23	−0.25 (−5.6%)	0.007 #	1.27/Large
	W	4.21 ± 0.12	4.31 ± 0.16	−0.10 (−2.4%)	0.113	0.67/Moderate
	B	4.26 ± 0.13	4.32 ± 0.17	−0.06 (−1.3%)	0.142	0.37/Small
	GK	4.43 ± 0.24	4.48 ± 0.16	−0.05 (−1.1%)	0.625	0.26/Small

Note: P—pivots, W—wingers, B—backs, GK—goalkeepers; ^#^ a nonparametric test was used to compare the groups.

**Table 3:** Power output (watts/kg body weight) and fatigue index (FI) in handball players as determined by the running-based anaerobic sprint test (RAST).

Indicator	Position	U19	U17	Mean Difference (%)	*p*-Value	Effect Size
P_max_ (Watts/kg body weight)	P	8.05 ± 0.84	7.02 ± 1.13	1.03 (12.8%)	0.060	1.00/Moderate
	W	9.66 ± 0.95	9.07 ± 1.47	0.59 (6.1%)	0.301	0.44/Small
	B	9.17 ± 0.95	8.96 ± 1.36	0.21 (2.3%)	0.499	0.17/Trivial
	GK	8.30 ± 1.15	7.89 ± 0.93	0.41 (4.9%)	0.440	0.42/Small
P_min_ (Watts/kg body weight)	P	5.70 ± 0.94	5.07 ± 0.91	0.63 (11.1%)	0.183	0.69/Moderate
	W	6.68 ± 0.64	6.13 ± 1.17	0.55 (8.3%)	0.218	0.52/Small
	B	6.57 ± 0.78	6.37 ± 0.99	0.2 (3.1%)	0.383	0.22/Small
	GK	5.47 ± 0.88	5.63 ± 0.90	−0.16 (−2.9%)	0.742	0.18/Trivial
P_av_ (Watts/kg body weight)	P	6.69 ± 0.77	5.88 ± 0.98	0.81 (12.1%)	0.088	0.90/Moderate
	W	7.98 ± 0.74	7.62 ± 1.05	0.35 (4.4%)	0.391	0.36/Small
	B	7.71 ± 0.81	7.57 ± 1.12	0.14 (1.9%)	0.574	0.14/Trivial
	GK	6.67 ± 1.05	6.75 ± 0.87	−0.08 (−1.2%)	0.874	0.08/Trivial
FI	P	29.24 ± 8.66	27.79 ± 7.02	1.44 (4.9%)	0.709	0.19/Trivial
	W	30.60 ± 5.11	32.71 ± 14.99	−2.11 (−6.9%)	0.702	0.16/Trivial
	B	28.27 ± 5.73	28.57 ± 7.68	−0.3 (−1.1%)	0.865	0.04/Trivial
	GK	34.17 ± 4.23	28.82 ± 6.36	5.35 (15.7%)	0.104	0.91/Moderate

Note: P—pivots; W—wingers; B—backs; GK—goalkeepers; FI—fatigue index.

**Table 4:** Absolute power (watts) produced in the players of various playing positions, recorded in RAST.

Indicator	Position	U19	U17	Mean Difference (%)	*p*-Value	Effect Size
P_max_ (Watts)	P	798.2 ± 125.4	641.8 ± 112.9	156.4 (19.6%)	0.017	1.33/Large
	W	807.5 ± 121.9	630.9 ± 121.5	176.6 (21.9%)	<0.001 ^#^	1.45/Large
	B	784.4 ± 98.2	701.2 ± 122.2	83.2 (10.6%)	0.005	0.73/Moderate
	GK	777.9 ± 102.0	641.1 ± 80.1	136.8 (17.6%)	0.008	1.59/Large
P_min_ (Watts)	P	561.5 ± 88.9	459.6 ± 65.5	101.9 (18.2%)	0.015	1.35/Large
	W	556.8 ± 62.9	425.9 ± 89.5	130.9 (23.5%)	0.001	1.57/Large
	B	563.1 ± 85.3	497.0 ± 79.8	66.0 (11.7%)	0.002	0.81/Moderate
	GK	511.4 ± 66.2	455.7 ± 65.1	55.6 (10.9%)	0.125	0.85/Moderate
P_av_ (Watts)	P	662.6 ± 100.9	534.7 ± 80.1	127.9 (19.3%)	0.011	1.44/Large
	W	666.4 ± 92.2	529.8 ± 86.6	136.6 (20.5%)	0.001 ^#^	1.55/Large
	B	661.0 ± 91.6	591.6 ± 96.1	69.5 (10.5%)	0.004	0.74/Moderate
	GK	623.8 ± 78.6	547.3 ± 64.9	76.5 (12.3%)	0.050	1.12/Moderate

Note: P—pivots; W—wingers; B—backs; GK—goalkeepers; ^#^ a nonparametric test was used to compare the groups.

**Table 5:** Internal load parameters of the subjects.

Indicator	Position	U19	U17	Mean Difference (%)	*p*-Value	Effect Size
HRmax [beats/min]	P	195.4 ± 8.72	199.1 ± 5.93	N/A	N/A	N/A
	W	196.4 ± 5.29	200.3 ± 6.7	N/A	N/A	N/A
	B	196.0 ± 10.13	195.6 ± 5.81	N/A	N/A	N/A
	GK	193.6 ± 5.03	199.4 ± 3.66	N/A	N/A	N/A
HRAnT [%HRmax]	P	91.8 ± 2.79	89.6 ± 4.18	2.20 (2.4%)	0.244	0.60/Small
	W	91.6 ± 3.20	91.0 ± 4.09	0.58 (0.6%)	0.720	0.15/Trivial
	B	89.8 ± 4.86	91.0 ± 3.26	−1.21 (−1.4%)	0.227	0.31/Small
	GK	90.1 ± 3.89	91.5 ± 2.93	−1.38 (−1.5%)	0.425	0.43/Small
HRAeT [%HRmax]	P	83.4 ± 1.63	82.2 ± 4.90	1.20 (1.4%)	0.770 ^#^	0.31/Small
	W	82.6 ± 4.09	84.5 ± 4.30	−1.88 (−2.3%)	0.292	0.44/Small
	B	80.8 ± 5.06	83.5 ± 3.83	−2.72 (−3.4%)	0.015	0.63/Moderate
	GK	82.6 ± 2.18	82.6 ± 3.99	−0.01 (−0.01%)	0.997	0/Trivial

Note: P—pivots; W—wings; B—backs; GK—goalkeeper; N/A—not appropriate; ^#^ a nonparametric test was used to compare the groups.
